# P-1860. Factors Associated with Readmission in Immunocompromised Hosts Receiving Outpatient Parenteral Antimicrobial Therapy

**DOI:** 10.1093/ofid/ofaf695.2029

**Published:** 2026-01-11

**Authors:** Jamila I Ranavaya, Nicole Leedy, Takaaki Kobayashi, Ryan P Mynatt, Evelyn Villacorta Cari, Ashley Logan, Armaghan-E Rehman Mansoor

**Affiliations:** University of Kentucky, Lexington, KY; University of Kentucky, Lexington, KY; University of Kentucky, Lexington, KY; University of Kentucky, Lexington, KY; University of Kentucky, Lexington, KY; University of Kentucky HealthCare, Lexington, Kentucky; University of Kentucky, Lexington, KY

## Abstract

**Background:**

OPAT provides a framework for safe administration of intravenous (IV) antimicrobials outside the hospital. Immunocompromised hosts are at risk for clinical events related to, or independent of OPAT that may result in hospital readmission. Our study evaluates outcomes and causes of OPAT readmission in patients with immune compromise (IC).

**Figure 1:**
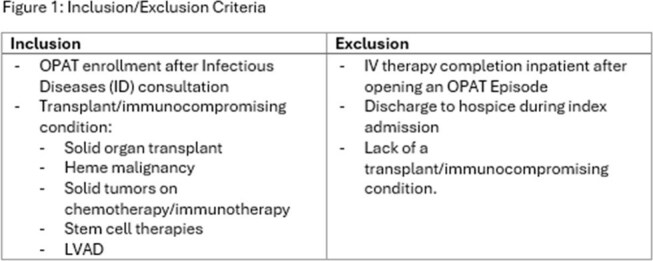
Inclusion/Exclusion Criteria

**Figure 2:**
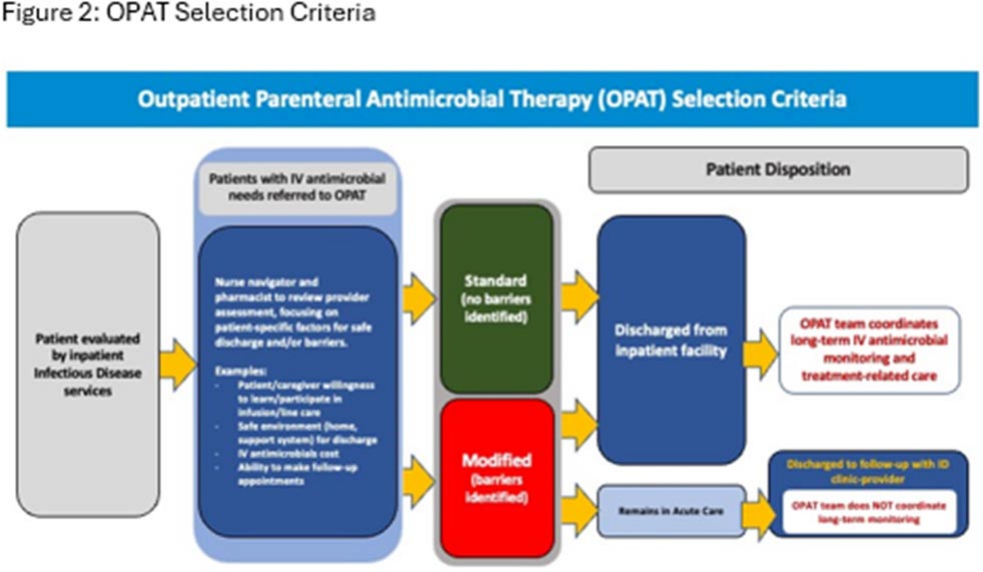
OPAT Selection Criteria

**Methods:**

This is a retrospective cohort study of patients admitted to University of Kentucky Healthcare, a tertiary referral center between June 2021 and December 2023. An OPAT episode was defined by the time during which a patient received IV therapy for a specific indication after hospital discharge (Figure 1). Patients were assigned to Standard vs Modified based on internal criteria (Figure 2). Outcome measures included proportion of patients with readmission within 90 days of discharge, number of readmissions, and cause of readmission and relation to OPAT services.

**Table 1:**
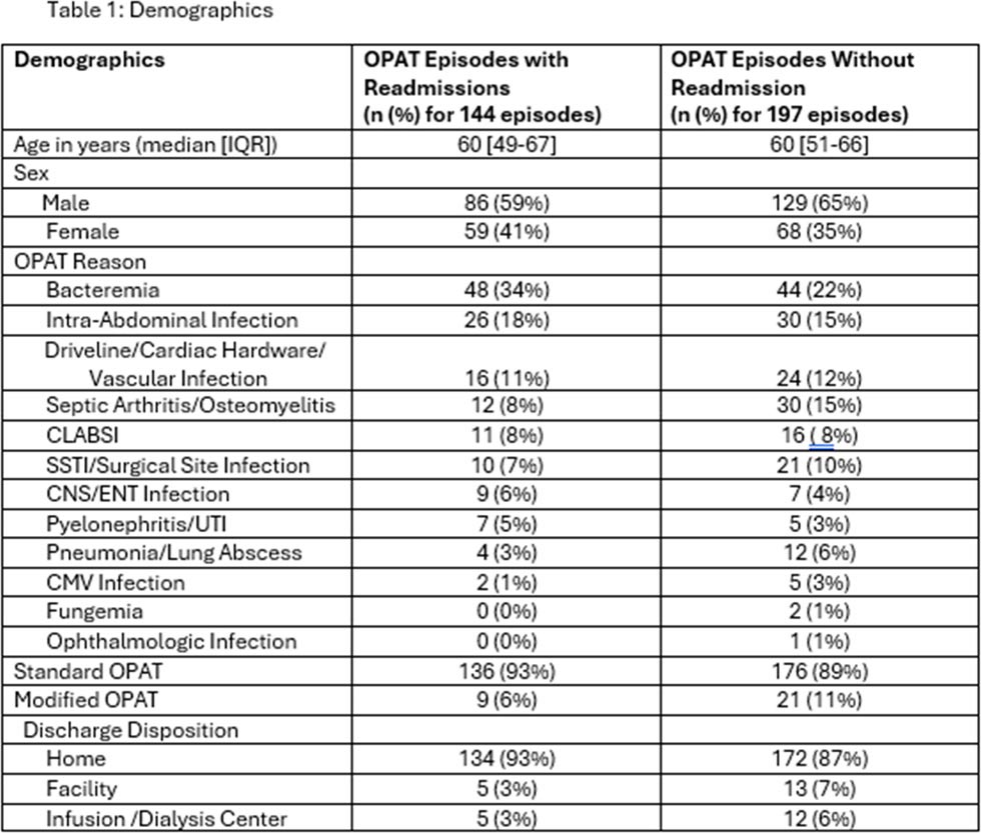
Demographics

**Table 2:**
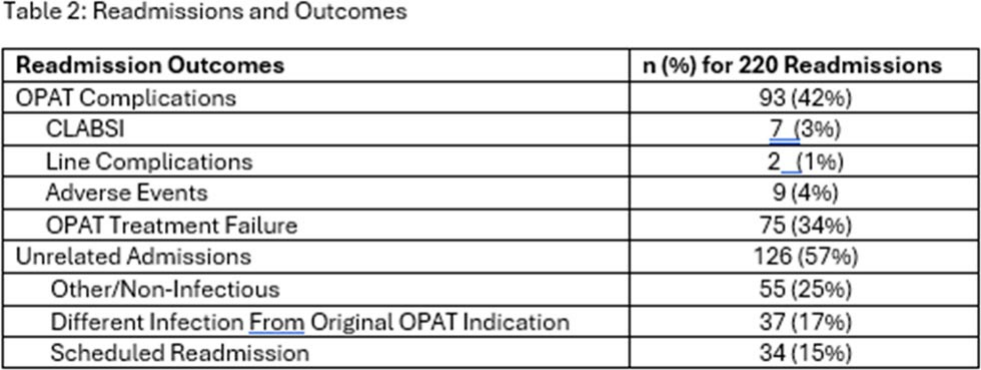
Readmissions and Outcomes

**Results:**

In the study period, 341 OPAT episodes occurred in 312 patients, of which 144 OPAT episodes (42%) had ≥ 1readmission within 90 days of discharge, with a total of 220 readmissions. Median age was 60 years (IQR 49- 67), with 59% male patients. The most common indications for OPAT were bacteremia (34%) and intra-abdominal infection (18%) (Table 1). Most readmissions were non-OPAT related (126/220, 57%), with other/non-infectious causes (55/220, 25%) as the largest in that subgroup. There was no significant difference in age and or sex between patients who had readmissions vs those who did not. The highest proportion of readmissions occurred in patients receiving OPAT for bacteremia (52%), followed by intra-abdominal infection (46%) and cardiac hardware/vascular infections (40%).

**Conclusion:**

In a cohort of immunocompromised hosts, a high rate of 90-day readmission following OPAT was recorded (42%), however most events were unrelated to OPAT. A significant proportion of unplanned readmissions were due to potential treatment failure or new infection, a finding that merits further evaluation into clinical factors that may predict risk of persistent or recurrent infection. OPAT continues to be a feasible method of transitioning parenteral therapy from the inpatient setting.

**Disclosures:**

All Authors: No reported disclosures

